# Inflammation Markers and HCC Aggressiveness

**DOI:** 10.37871/jbres2093

**Published:** 2025-04-28

**Authors:** Brian Irving Carr, Rossella Donghia

**Affiliations:** 1 Liver Transplant Institute, Inonu University Faculty of Medicine, 44280, Malatya, Turkey; 2 National Institute of Gastroenterology - IRCCS “Saverio de Bellis”, Italy

**Keywords:** HCC, MTD, PVT, Albumin, CRP, HALP, Survival

## Abstract

**Background:**

Inflammation is thought to be important in the development and progression of Hepatocellular Carcinoma (HCC), but which inflammatory indices are more useful in clinical practice is not clear.

**Aims:**

Several inflammatory indices were examined with respect to Maximum Tumor Diameter (MTD), Portal Vein Thrombosis by Tumor (PVT) and survival.

**Results:**

Serum C-Reactive Protein (CRP) and Platelet Lymphocyte Ratio (PLR) each significantly increased with increasing MTD as did percent patients with higher Glasgow index; whereas albumin levels and HALP (Hemoglobin, Albumin, Lymphocytes and Platelets) score decreased, as expected. Lower (abnormal) albumin was associated with higher alpha-fetoprotein and percentage PVT patients. Cox proportional hazard models for death showed a significant protective effect for albumin, whereas CRP and the Glasgow score had significant Hazard Ratios (HR) for death, but neither PLR nor the HALP score had significant HRs. Kaplan-Meier analysis showed reduced survival at each MTD for patients with low versus high albumin levels. All parameters were significantly different for presence versus absence of PVT, but only albumin and CRP had significance in Cox proportional hazard models for death in patients with PVT and only albumin for patients with high alpha-fetoprotein.

**Conclusion:**

Each inflammation parameter worsened with increase in MTD, but only albumin and CRP (and the Glasgow index) were significant for survival in the total cohort. Only serum albumin had significance for survival in patients with PVT or high alpha-fetoprotein.

## Introduction

Inflammation has been considered as mechanistically important for the development of cancer since Virchow [1]. It is thought to involve the tumor microenvironment [2]. Hepatocellular Carcinoma (HCC) most commonly develops on the basis of several chronic inflammatory diseases, including hepatitis B or hepatitis C, Metabolic dysfunction-Associated Steatohepatitis (MASH) and chronic alcoholism [3]. Several inflammation markers have been used in HCC patient assessment, as indices of prognosis and even tumor aggressiveness [4–7] and include C-Reactive Protein (CRP), albumin, the combination of CRP and albumin (Glasgow Index), Erythrocyte Sedimentation Rate (ESR), Platelet To Lymphocyte Ratio (PLR), Hemoglobin, Albumin, Lymphocyte, Platelet (HALP) score [8,9] and the liver inflammation parameters AST and ALT levels.

Given the increasing numbers of inflammation scores available to clinical practitioners, this study was conducted on a large HCC dataset, to compare HALP, Glasgow and several commonly available single parameter inflammation markers for their usefulness in HCC patient evaluation.

## Methods

### Clinical

A clinical HCC database with 6759 patients was further examined, that we have previously interrogated [10]. The data was collected from the Italian Liver Cancer (ITA.LI.CA) database of HCC patients. Patients had routine hematology, liver function tests, Child-Pugh score constituents (albumin, total bilirubin, prothrombin time, and presence/absence of ascites and encephalopathy) and AFP levels recorded on their initial clinic visit, as well as baseline tumor characteristics of maximum tumor diameter, tumor number, and presence/absence of macroscopic malignant portal vein thrombosis by Computed Axial Tomography (CAT) scan evaluation. All patients were diagnosed according to EASL criteria. Macroscopic portal vein thrombosis by HCC was assessed by presence of heterogeneous contrast-enhanced portal vein thrombosis with enlargement (>23 mm) in proximity to vascular HCC.

## Statistical Analysis

Patients’ parameters are reported as median and Interquartile Range (IQR) for continuous variables, and as frequency and percentage (%) for categorical variables. Comparisons between independent groups (MTDs categories) was performed with Wilcoxon rank-sum test for number of groups of 2 (PVT(+) *vs* PVT (−)), while Kruskal-Wallis test for a number of groups of 3 for continuous variables (MTD categories or combination of MTD and albumin), while for categorical variables Chi-Square or Fisher was used. The associations between singles parameters and risk of mortality was tested with Cox model, with Hazard Ratio (HR) as estimate with Confidence Interval of 95% (C.I. 95%). Survival probability on combination of liver parameters was explored using a non-parametric Kaplan-Meier method. The equality of survival curves was analyzed using the log-rank test. To test the null hypothesis of non-association, the two-tailed probability level was set at 0.05. Analyses were conducted with Stata Statistical Software: Release 18, StataCorp, 2023, StataCorp LLC.: College Station, TX, USA.

## Results

Several serum parameters of liver and systemic inflammation (albumin, CRP, PLR, HALP score, Glasgow score) were examined across MTD categories (<5 cm/5–10 cm/>10 cm), as shown in the table 1. Median levels of CRP and PLR each significantly increased with increasing MTD (83.39, 93.65, and 127.91) statistically significant; whereas levels of albumin and HALP score decreased, as expected (*p* < 0.05). Glasgow score level increased with increase in MTD. These results showed that parameters for both liver and systemic inflammation increased with MTD increase. These findings were supported by the results of dichotomizing the parameters, as shown in the bottom 2 rows for each parameter in table 1. The percentage of patients with the worse inflammation parameter levels (albumin <3.50 g/dL, PLR >150, CRP, >1.0 mg/dL, HALP score <42.0 and Glasgow score 1 (versus 0) all increased with increase in MTD group.

Since serum albumin levels reflect liver synthetic dysfunction, poor nutrition from cancer, systemic inflammation (it is a negative acute phase protein) and is widely used (together with CRP levels) as the Glasgow index of GI cancer prognosis, we compared patients in each MTD group who were dichotomized for low (abnormal) versus high albumin levels (Table 2). At each MTD, patients with low serum albumin levels of <3.5g/dL had reduced levels of hemoglobin (12.20), platelets (84.00) and HALP inflammation score, as well as increased tumor parameters AFP and percent of patients with PVT.

**Figure 1:**
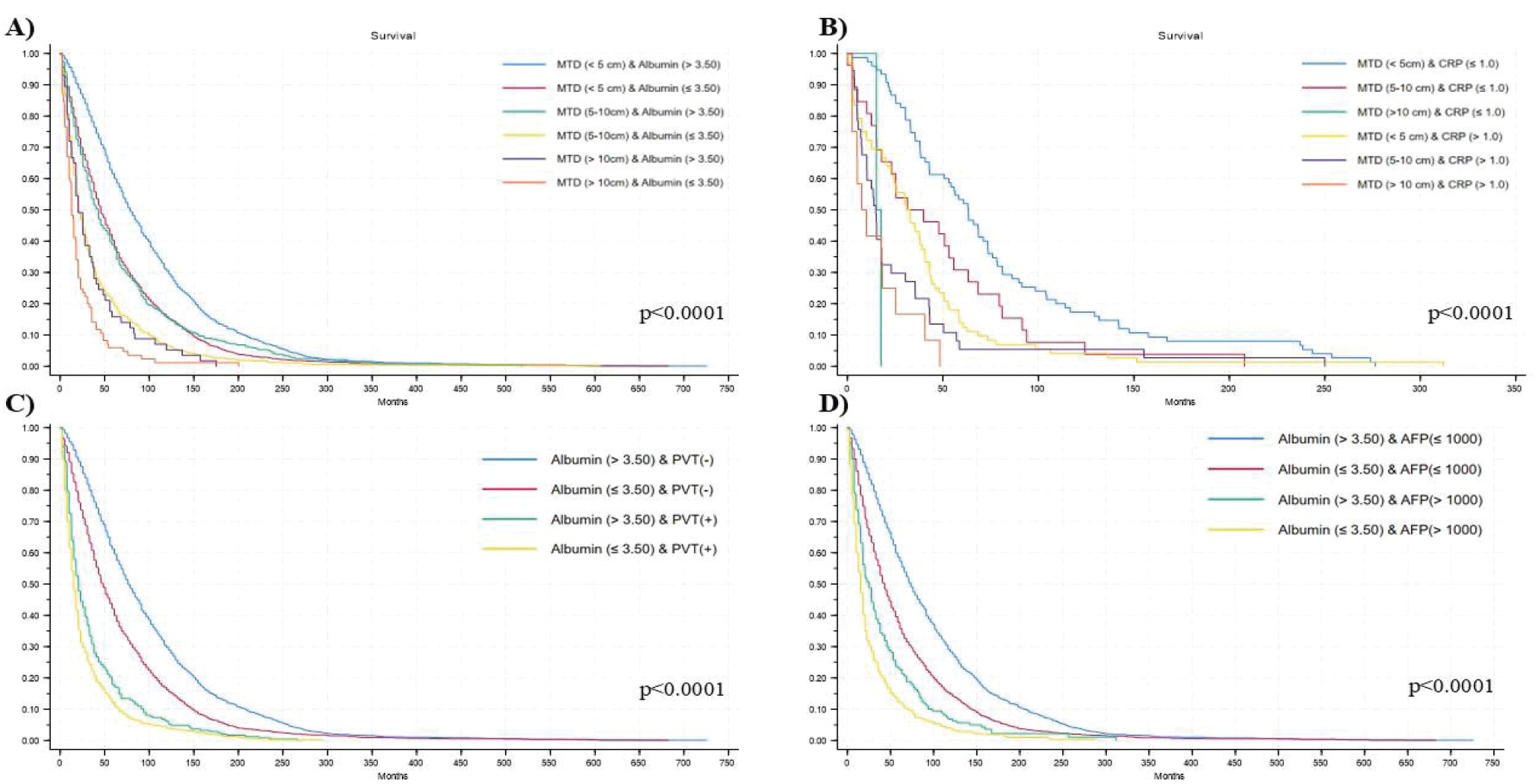
Kaplan-Meier survival plot on patients with different MTDs, dichotomized by albumin (A), or by CRP or C-reactive protein (B); on patients with PVT, dichotomized by albumin (C); and on patients with elevated AFP, dichotomized by albumin (D).

Using Kaplan-Meier (KM) analysis for survival, patients in each MTD had lower survival if they had lower compared with higher albumin levels (Figure 1A), as well as higher compared with lower CRP levels (Figure 1B) with statistical differences between groups (*p* < 0.0001).

Cox proportional hazards models for death applied to the clinical parameters in the total cohort, showed a significant protective effect for albumin, both as continuous and as categorical variables (HR = 0.61 and HR = 0.67, *p* < 0.001) (Table 3). CRP had a significant Hazard Ratio (HR) on death in both continuous and categorical variables (HR of 1.05 and 2.12, respectively, *p* < 0.001), as did the Glasgow score (HR = 1.74 and 1.73, *p* < 0.001), but neither the PLR nor the HALP score was significant for death as continuous variables (*p* > 0.05).

The inflammation parameters were also examined for presence or absence of macroscopic tumor PVT (Table 4), in a similar manner to the MTD analysis (Table 1). All parameters were significantly different for presence compared to absence of PVT, with albumin decreasing in PVT patients (3.30 *vs* 3.60, *p* < 0.0001), and with large increases in CRP (2.00 *vs* 1.00, *p* < 0.0001) and Glasgow score grade 1 (61.90% *vs* 42.00%, *p* < 0.001).

The patients with PVT had survival results calculated using the KM method that showed better survival for the PVT patient subset who had higher (normal) serum albumin levels (Figure 1C).

Cox proportional hazard models for death based on clinical parameters in PVT patients were then calculated.

The results showed that serum albumin was significantly protective in both continuous and categorical analyses (Table 5). However, CRP only had a significant HR (HR = 1.05, *p* = 0.001, 1.02 to 1.08 95% C.I.) in continuous but not in categorical analysis (HR = 1.57, *p* = 0.09, 0.93 to 2.65 95% C.I.). However, PLR, HALP and Glasgow scores did not have significant HRs as either continuous or categorical variables. In patients with PVT.

A KM survival curve was then calculated for different combinations between albumin and AFP level categories. Lower survival probability was shown in patients with low albumi (≤3.50) and high AFP (>1000), while the opposite condition showed the best survival probability, with statistical differences between categories (*p* < 0.0001) (Figure 1D).

**Figure 2:**
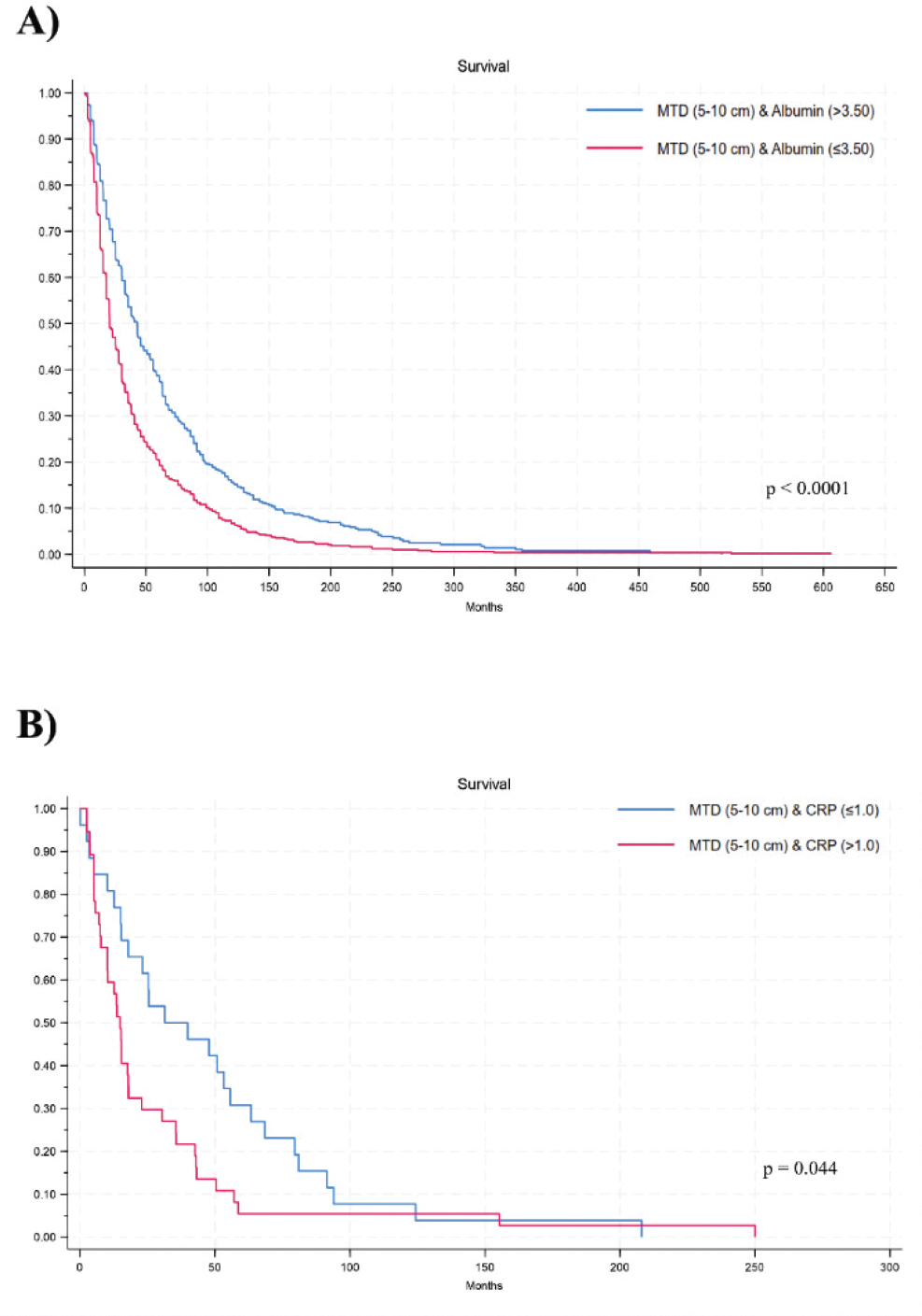
Kaplan-Meier survival plot on patients with different MTDs, dichotomized by albumin (A), and or by CRP or C-reactive protein (B).

Furthermore, when stratifying for some MTD categories by albumin (Figure 2A) or CRP (Figure 2B), considering comparison of the 2-by-2 survival curves, differences remain significant (*p* < 0.05).

Cox proportional hazard models for death based on clinical parameters for patients with AFP > 1000 levels were also calculated, but only albumin levels were significant and protective (HR = 0.68, *p* < 0.001, 0.59 to 0.79 95% C.I.) (Table 6). None of the other inflammation parameters had significant HRs (*p* < 0.05) in the high serum AFP subset.

## Discussion

The main results of this study were that all inflammation parameters had worsening values with increase in Maximum Tumor Diameter (MTD), with associated increases in serum CRP (C-Reactive Protein) and PLR (Platelet-Lymphocyte Ratio), level of Glasgow index (CRP plus Albumin), and decreases in serum albumin and HALP (Hemoglobin, Albumin, Lymphocytes and Platelets) score. Patients with reduced albumin also had higher levels of serum alpha-fetoprotein and percentage of patients with PVT (portal vein tumor thrombosis), compared to patients with high (normal) albumin levels. However, the parameters differed with respect to survival. Cox proportional hazard models for death showed a significant protective effect for albumin, whereas C-Reactive Protein (CRP) and the Glasgow score had significant Hazard Ratios (HR) for death, but not PLR nor the HALP score. Furthermore, Kaplan-Meier analysis showed decreased survival at each MTD (maximum tumor diameter) level for patients with low versus high albumin levels. For patients with PVT, all parameters were significantly different for presence versus absence of PVT, but only albumin had significance in Cox proportional hazard models for death. Similarly, for patients with elevated serum AFP levels, only albumin level was significantly associated with increased survival time. None of the other inflammation parameters had significant HRs. Thus, all parameter levels were worse with increase in MTD or development of PVT or elevated serum AFP levels. However, with respect to death, only albumin and CRP were significant in the 3 MTD groups. For patients with PVT, only albumin (and to some extent CRP) was significant and protective. Similarly for patients with elevated serum AFP levels, only albumin was significantly associated with survival.

The tumor microenvironment is considered to be very important in both carcinogenesis and the development of tumor aggressiveness traits [11], namely growth, invasion and metastasis [12]. It consists of cellular and non-cellular components such as cytokines, chemokines, growth factors and immune mediators, all of which contribute to tumor-associated inflammation, which itself is considered to be a 2-way process. This is in part because inflammation influences albumin synthesis and catabolism, and albumin can in turn act as a scavenger of inflammation-associated reactive oxygen species [13].

Albumin has several physiological functions in the blood and its levels have been described as both an inflammation [14–18] and a prognosis marker in HCC patients [7,19–23]. Albumin may actively participate in HCC growth control mechanisms [24–26], possibly through its ability to bind reactive oxygen species and so modulate inflammation [27] and has been shown to be an index of hepatocellular differentiation [28]. Serum albumin and AFP levels have an inverse relationship in the transition from embryonic to post-natal human life and this reciprocal relationship has also been reported in adult HCC patients [29]. The proposed mechanisms underlying this reciprocity have been much studied and may occur at the transcriptional level [30–32]. Furthermore, detection of albumin mRNA in peripheral blood has been proposed to be a marker for circulating HCC cells [33,34].

Several inflammation scores were examined in this study, including single parameter albumin and CRP, and combination parameter PLR, Glasgow and HALP scores. Both Glasgow and HALP contain albumin as one of the prognosis parameters. While the single and combination parameters all increased with increase in MTD and by the presence of PVT, only single parameter albumin and CRP and Glasgow score (containing both albumin and CRP) were consistently significant (and protective in the case of single parameter albumin) in both continuous and categorical analyses, in the Cox proportional hazard models for death in the total cohort. However, Cox proportional hazard model for death in patients with PVT, showed that only single parameter albumin was significant (and protective) in both continuous and categorical models, in this important group of poor prognosis HCC patients. For patients with high AFP levels which also confer a poor prognosis, only albumin, but not CRP had a statistically significant effect on survival (Figures 1C,1D).

Thus, albumin levels were significantly lower in patients with higher MTD, presence of PVT or presence of elevated AFP levels, all 3 of which are known poor prognosis factors. However, normal albumin had a protective effect on death in presence of all these factors. Since albumin has been shown experimentally to antagonize both growth and migration (a metastasis precursor), it is possible that albumin exerts these effects in patients. Contrariwise and more likely, growing and aggressive HCCs are able to depress the synthesis or increase the breakdown of serum albumin, as a result of which it loses its HCC inhibitory properties.

Yet inspection of tables 1 (MTD) and 4 (PVT) shows that the differences in inflammation parameter in relation to tumor property are only relative at best. Thus, in table 1, the largest MTD group has only 57.79 percent of patients with low albumin and only 59.26 percent of patients with high CRP and only 50.65 percent of patients with low HALP score (low albumin and HALP and high CRP are indicators of inflammation). Similarly in table 4, for patients with PVT, only 67.62 percent have low albumin, 64.76 percent have high CRP and only 52.19 percent have a low HALP score. This means that either these inflammation indices are an insufficient explanation for the poorer prognosis tumors, or alternatively, there might be 2 different pathways that mediate these poorer prognosis HCC characteristics, one associated with increased inflammation indices and the other not. Support for the second explanation comes from the observation in tables 1,4, that the inflammation indices seem to change in tandem, that is, low albumin, low HALP and high CRP seem to change together, suggesting an overall change of more or of less inflammation. Multiple inflammation-associated factors have been described [11–13] that could be mechanistic candidates for these 2 pathways. In addition, for patients within the same MTD, or AFP or PVT group, the level of albumin (MTD, AFP, PVT) or CRP (MTD) results in significant differences in survival (Figure 1). Thus, not only do albumin levels relate to the presence of these aggressiveness characteristics, but once they are present, albumin levels significantly relate to prognosis.

## Figures and Tables

**Table 1:** Comparison of dichotomized parameters in MTD groups (<5 cm/5–10 cm/>10 cm) in total cohort.

Parameters*	MTD (cm)			*p* ^
	< 5cm (*n* = 5202) (76.96%)	5–10cm (*n* = 1331) (19.69%)	> 10cm (*n* = 226) (3.34%)	
Albumin (g/dL)	3.60 (0.80)	3.50 (0.90)	3.40 (0.70)	0.0001 ^†^
Albumin (g/dL) (%)				<0.001
< 3.50	2237 (48.40)	665 (54.64)	115 (57.79)	
≥ 3.50	2385 (51.60)	552 (45.36)	84 (42.21)	
PLR	83.39 (63.48)	93.65 (84.26)	127.91 (97.06)	0.0001 ^†^
PLR (%)				<0.001
< 150.0	902 (85.90)	199 (74.53)	37 (60.66)	
≥ 150.0	148 (14.10)	68 (25.47)	24 (39.34)	
CRP (mg/L)	1.00 (1.01)	1.56 (2.00)	3.00 (6.28)	0.0005 ^†^
CRP (mg/L) (%)				<0.001
≤ 1.0	270 (65.53)	46 (43.81)	11 (40.74)	
> 1.0	142 (34.47)	59 (56.19)	16 (59.26)	
HALP Score (%)				<0.001
≤ 42.0	323 (32.96)	113 (45.20)	34 (59.65)	
> 42.0	657 (67.04)	137 (54.80)	23 (40.35)	
Glasgow Score (%)				0.05
0	234 (57.97)	47 (44.76)	11 (44.00)	
1	176 (42.93)	58 (55.24)	14 (56.00)	

* As median and Interquartile Range (IQR) for continuous variables, and frequency and percentage (%) for categorical.

^ Chi-Square Test

† Kruskal-Wallis rank test.

**Abbreviations:** MTD: Maximum Tumor Diameter; PLR: Platelet-Lymphocyte Ratio; CRP: C-Reactive Protein; HALP: Hemoglobin Albumin Lymphocytes and Platelets; Glasgow score, CRP plus albumin.

**Table 2:** Comparison of parameters in MTD/Albumin groups in patients with total bilirubin ≤ 1.50 mg/dL.

#Parameters *	MTD (cm) & Albumin (g/dL)						*p* ^
	MTD (<5) & Albumin (>3.5) (*n* = 385)	MTD (<5) & Albumin (≤3.5) (*n* = 1033)	MTD (5–10) & Albumin (>3.5) (*n* = 109)	MTD (5–10) & Albumin (≤3.5) (*n* = 300)	MTD (>10) & Albumin (>3.5) (*n* = 16)	MTD (>10) & Albumin (≤3.5) (*n* = 50)	
^#^AST	1.60 (2.00)	2.00 (1.81)	1.90 (2.00)	2.45 (2.50)	4.51 (26.00)	2.95 (4.65)	0.0001
^#^ALT	1.20 (1.46)	1.50 (1.57)	1.24 (1.60)	2.00 (2.10)	2.00 (22.68)	2.00 (4.16)	0.0001
^#^ALKP	1.00 (0.45)	1.09 (0.70)	1.00 (0.68)	1.40 (1.00)	2.25 (139.00)	2.00 (2.22)	0.0001
^#^GGT	1.98 (2.79)	1.80 (2.50)	2.80 (4.75)	2.87 (3.77)	10.75 (169.14)	4.00 (3.34)	0.0001
^#^Total Bilirubin	2.00 (0.80)	2.50 (1.90)	2.10 (1.40)	2.57 (2.36)	1.99 (1.08)	2.45 (1.78)	0.0001
Hemoglobin (g/dL)	13.50 (2.90)	12.20 (2.60)	13.90 (3.00)	12.00 (2.90)	12.90 (4.00)	13.00 (3.60)	0.0001
Platelets (10^9^/L)	89.00 (60.00)	84.00 (58.00)	107.00 (69.00)	102.00 (81.00)	153.00 (229.00)	145.00 (130.00)	0.0001
AFP (ng/mL)	11.20 (42.50)	17.00 (116.00)	38.50 (556.20)	81.50 (733.65)	140.0 (271.00)	216.00 (5170.00)	0.0001
PVT (Yes) (%)	47 (12.37)	226 (22.16)	30 (28.04)	116 (39.46)	5 (31.25)	29 (58.00)	<0.001
HALP Score							<0.001
≤ 42.0	10 (13.51)	80 (41.03)	4 (14.29)	29 (60.42)	2 (66.67)	12 (63.16)	
> 42.0	64 (86.49)	115 (58.97)	24 (85.71)	19 (39.58)	1 (33.33)	7 (36.84)	

* As Median and Interquartile Range (IQR) for continuous variables; and frequency and percentage (%) for categorical.

^ Kruskal-Wallis rank test

ψ Chi-Square or Fisher test when necessary.

# X-old Upper Limit of Normal.

**Abbreviations:** MTD: Maximum Tumor Diameter (cm); Albumin (g/dL); AST, Aspartate Transferase (IU/mL); ALT, Alanine Transaminase (IU/mL); ALKP, alkaline phosphatase (IU/L); GGT, Gamma-Glutamyl Transferase (IU/L); Total Bilirubin (mg/dL); AFP, Alpha-Fetoprotein; PVT, Portal Vein Thrombosis; HALP: Hemoglobin, Albumin, Lymphocytes and Platelets.

**Table 3:** Cox proportional hazard models for death on clinical parameter in the total cohort.

Parameters *	HR	se (HR)	*p*	95% C.I.
Albumin (g/dL)	0.61	0.02	<0.001	0.58 to 0.65
Albumin (g/dL) (%)				
≤ 3.50 *[Ref.]*	--	--	--	--
> 3.50	0.64	0.02	<0.001	0.60 to 0.68
PLR	0.99	0.001	0.31	0.99 to 1.00
PLR (%)				
≤ 150.0 *[Ref.]*	--	--	--	--
> 150.0	1.05	0.11	0.64	0.86 to 1.28
CRP (mg/L)	1.05	0.01	<0.001	1.03 to 1.07
CRP (mg/L) (%)				
≤ 1.0 *[Ref.]*	--	--	--	--
> 1.0	2.12	0.29	<0.001	1.62 to 2.79
HALP Score	0.99	0.001	0.25	0.99 to 1.00
HALP Score (%)				
≤ 42.0 *[Ref.]*	--	--	--	--
> 42.0	0.78	0.06	0.003	0.66 to 0.91
Glasgow Score	1.74	0.22	<0.001	1.35 to 2.24
Glasgow Score (%)				
0 *[Ref.]*	--	--	--	--
1	1.73	0.25	<0.001	1.31 to 2.29

* As frequency and percentage (%) for categorical.

^ Chi-Square test.

**Abbreviations:** HR: Hazard Ratio; se (HR): Standard Error of HR; 95% C.I.; Confidential Interval at 95%; PLR: Platelet-Lymphocyte Ratio; CRP: C-Reactive Protein; HALP: Hemoglobin, Albumin, Lymphocytes and Platelets; Glasgow score, CRP plus albumin.

**Table 4:** Comparison of dichotomized parameters in PVT groups (Yes vs No) in total cohort.

Parameters *	PVT		*p* ^
	No (*n* = 5513) (83.44%)	Yes (*n* = 1094) (16.56%)	
Albumin (g/dL) (%)	3.60 (0.80)	3.30 (0.80)	<0.0001
Albumin (g/dL) (%)			<0.001
≤ 3.50	2288 (46.22)	685 (67.62)	
> 3.50	2662 (53.78)	328 (32.38)	
PLR	84.92 (64.96)	97.19 (76.53)	<0.0001
PLR (%)			0.003
≤ 150.0	952 (83.95)	179 (75.85)	
> 150.0	182 (16.05)	57 (24.15)	
CRP (mg/L)	1.00 (1.03)	2.00 (3.20)	<0.0001
CRP (mg/L) (%)			<0.001
≤ 1.0	287 (65.98)	37 (35.24)	
> 1.0	148 (34.02)	68 (64.76)	
HALP Score (%)			<0.001
≤ 42.0	349 (33.21)	119 (52.19)	
> 42.0	702 (66.79)	109 (47.81)	
Glasgow Score (%)			<0.001
0	250 (58.00)	40 (38.10)	
1	181 (42.00)	65 (61.90)	

* As Median and Interquartile Range (IQR) for continuous variables, and frequency and percentage (%) for categorical.

^ Chi-Square test

† Wilcoxon rank-sum test.

**Abbreviations:** PVT: Portal Vein Thrombosis; PLR: Platelet-Lymphocyte Ratio; CRP: C-Reactive Protein; HALP: Hemoglobin, Albumin, Lymphocytes and Platelets; Glasgow score, CRP plus albumin.

**Table 5:** Cox proportional hazard models for death on clinical parameter in PVT (+) sub-cohort.

Parameters *	HR	se (HR)	*p*	95% C.I.
Albumin (g/dL)	0.71	0.04	<0.001	0.63 to 0.81
Albumin (g/dL) (%)				
≤ 3.50 *[Ref.]*	--	--	--	--
> 3.50	0.79	0.06	0.002	0.68 to 0.92
PLR	0.99	0.001	0.78	0.99 to 1.00
PLR (%)				
≤ 150.0 *[Ref.]*	--	--	--	--
> 150.0	1.22	0.23	0.29	0.84 to 1.77
CRP (mg/L)	1.05	0.01	0.001	1.02 to 1.08
CRP (mg/L) (%)				
≤ 1.0 *[Ref.]*	--	--	--	--
> 1.0	1.57	0.42	0.09	0.93 to 2.65
HALP Score	0.99	0.001	0.19	0.99 to 1.00
HALP Score (%)				
≤ 42.0 *[Ref.]*	--	--	--	--
> 42.0	0.77	0.12	0.12	0.56 to 1.06
Glasgow Score	1.55	0.39	0.08	0.95 to 2.55
Glasgow Score (%)				
0 *[Ref.]*	--	--	--	--
1	1.36	0.37	0.25	0.81 to 2.31

* As frequency and percentage (%) for categorical.

^ Chi-Square test.

**Abbreviations:** HR: Hazard Ratio; se (HR): Standard Error of HR; 95% C.I.; Confidential Interval at 95%; PLR: Platelet-Lymphocyte Ratio; CRP: C-Reactive Protein; HALP, Hemoglobin, Albumin, Lymphocytes and Platelets; Glasgow score, CRP plus albumin.

**Table 6:** Cox proportional hazard models for death on clinical parameters in patients with AFP>1000. IU/mL.

Parameters *	HR	se (HR)	*p*	95% C.I.
Albumin (g/dL)	0.68	0.05	<0.001	0.59 to 0.79
Albumin (g/dL) (%)				
≤ 3.50 *[Ref.]*	--	--	--	--
> 3.50	0.71	0.07	<0.001	0.58 to 0.86
PLR	1.00	0.001	0.95	0.99 to 1.00
PLR (%)				
≤ 150.0 *[Ref.]*	--	--	--	--
> 150.0	1.28	0.32	0.33	0.78 to 2.10
CRP (mg/L)	1.04	0.02	0.08	0.99 to 1.08
CRP (mg/L) (%)				
≤ 1.0 *[Ref.]*	--	--	--	--
> 1.0	1.87	0.64	0.07	0.95 to 3.67
HALP Score	0.99	0.002	0.22	0.99 to 1.00
HALP Score (%)				
≤ 42.0 *[Ref.]*	--	--	--	--
> 42.0	0.69	0.16	0.11	0.44 to 1.08
Glasgow Score	1.41	0.44	0.27	0.77 to 2.59
Glasgow Score (%)				
0 *[Ref.]*	--	--	--	--
1	1.42	0.55	0.36	0.67 to 3.04

* As frequency and percentage (%) for categorical.

^ Chi-Square test.

**Abbreviations:** HR: Hazard Ratio; se (HR): Standard Error of HR; 95% C.I.: Confidential Interval at 95%; PLR: Platelet-Lymphocyte Ratio; CRP: C-Reactive Protein; HALP: Hemoglobin, Albumin, Lymphocytes and Platelets; Glasgow score, CRP plus albumin.

## Data Availability

The data are available on request to the authors, as they are not publicly available on legal grounds. However, all analyzed data were included in the article. Any further enquiries should be directed to the corresponding author.

## Abbreviations

HCC: Hepatocellular Carcinoma
MTD: Maximum Tumor Diameter
PVT: Portal Vein Thrombosis
CT: Computed Axial Tomography
AFP: Alpha-Fetoprotein
WBC: White Blood Count
T.Bil: Total Bilirubin
AST: Aspartate Amino Transferase
ALT: Alanine Aminotransferase
ALKP: Alkaline Phosphatase
GGT: Gamma Glutamyl Transpeptidase
CRP: C-Reactive Protein
PLR: Platelet Count to Lymphocyte Ratio
HALP: Hemoglobin, Albumin, Lymphocyte, Platelet Score
HR: Hazard Ratio; KM: Kaplan-Meier
